# Emerging Pharmacotherapies for Diabetic Macular Edema

**DOI:** 10.1155/2012/548732

**Published:** 2012-02-26

**Authors:** Golnaz Javey, Stephen G. Schwartz, Harry W. Flynn

**Affiliations:** ^1^Department of Ophthalmology, Baylor College of Medicine, 7200-B Cambridge Street, Houston, TX 77030, USA; ^2^Department of Ophthalmology, Bascom Palmer Eye Institute, University of Miami Miller School of Medicine, 311 9th Street North, No. 100, Naples, FL 34102, USA; ^3^Department of Ophthalmology, Bascom Palmer Eye Institute, University of Miami Miller School of Medicine, 900 Northwest 17th Street, Miami, FL 33136, USA

## Abstract

Diabetic macular edema (DME) remains an important cause of visual loss in patients with diabetes mellitus. Although photocoagulation and intensive control of systemic metabolic factors have been reported to achieve improved outcomes in large randomized clinical trials (RCTs), some patients with DME continue to lose vision despite treatment. Pharmacotherapies for DME include locally and systemically administered agents. We review several agents that have been studied for the treatment of DME.

## 1. Introduction

Diabetic macular edema (DME) is one of the most common causes of visual loss in patients with diabetes mellitus [1]. The pathophysiology of DME involves dilated capillaries, retinal microaneurysms, and loss of pericytes, with eventual impairment of the blood-retinal barrier (BRB) [2]. Breakdown of the BRB results in fluid leakage into the extracellular space, which disrupts macular structure and function on a cellular level [3, 4]. A technique for visualizing molecules leaked through the outer BRB in a diabetic rodent model has recently been described, which should increase our understanding of this process [5].

This leakage may be analyzed in terms of physical forces [6]. Starling's Law states that the net flow of fluid across a vessel wall is increased by hydrostatic pressure within the lumen of the vessel and decreased by oncotic pressure within the lumen. In diabetic patients, hydrostatic pressure may be increased because of systemic hypertension and retinal ischemia, increasing the likelihood of exudation. This problem is exacerbated because increased hydrostatic pressure may lead to dilatation and tortuosity of retinal arterioles, capillaries, and venules, which increases vessel wall tension and further disruption of the BRB according to LaPlace's Law [7]. Other factors may also contribute to this edema, such as osmotic stress leading to Muller cell swelling, such as that reported with retinal detachment [8].

The pathogenesis of DME is at this time poorly defined, but is believed to involve angiogenesis, inflammation, and oxidative stress [9]. Hyperglycemia is reported to lead to capillary endothelial damage and alterations in leukocyte function [10]. In addition, hyperglycemia has been reported to activate oxidative stress agents, such as advanced glycation endproducts and the protein kinase C (PKC) pathway [11]. Various inflammatory mediators appear to play a role in promoting DME, including vascular endothelial growth factor (VEGF) [12], placental growth factor (PlGF) [13], and hepatocyte growth factor (HGF) [14].

The Wisconsin Epidemiologic Study of Diabetic Retinopathy (WESDR) reported that approximately 14% of patients with type 2 diabetes developed DME over a 10-year period [15]. More recently, the 10-year incidence of DME in a Spanish population of patients with type 1 diabetes was reported as approximately 11% [16]. Reported risk factors for diabetic retinopathy and DME include duration of diabetes, as well as the severity of hyperglycemia, hypertension, and hyperlipidemia [17].

Intensive control of systemic factors, including blood sugar, blood pressure, and serum lipids, has been reported to reduce complications of diabetic retinopathy in patients with type 1 [18] and type 2 [19] diabetes. Macular photocoagulation was demonstrated as a treatment for clinically significant macular edema (CSME) by the Early Treatment Diabetic Retinopathy Study (ETDRS) in 1985 [20]. Newer clinical trials using intravitreal pharmacotherapies have reported many favorable outcomes. The current paper will review the literature and various randomized clinical trials (RCTs) on emerging pharmacotherapies for the treatment of DME.

## 2. Ocular Agents

### 2.1. Corticosteroids

**Figure 1:**
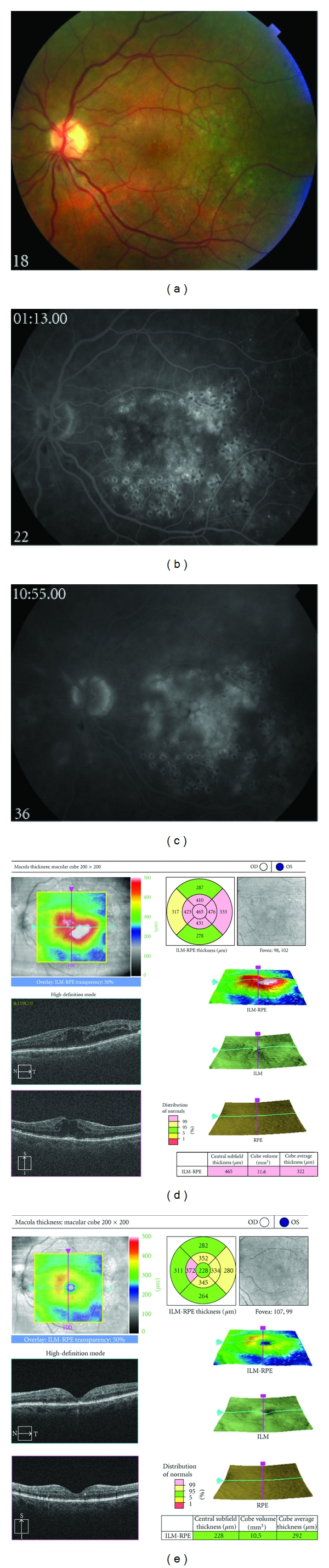
(a) Fundus photograph, left eye, of a patient with persistent diabetic macular edema following focal/grid photocoagulation. (b) Early phase fluorescein angiograph, left eye, demonstrating abnormal hyperfluorescence in the macula. (c) Late phase fluorescein angiograph, left eye, demonstrating profuse leakage consistent with angiographic macular edema. (d) Spectral domain optical coherence tomograph, left eye, demonstrating cystoid macular edema. (e) Following treatment with intravitreal triamcinolone acetonide, 4 mg in 0.1 mL, spectral domain optical coherence tomography demonstrates marked improvement in cystoid macular edema.

Corticosteroids may have multiple mechanisms of action in the treatment of DME. In addition to their anti-inflammatory properties, corticosteroids have been reported to reduce the activity of VEGF [21]. Intravitreal triamcinolone acetonide (IVTA) has been reported for the treatment of DME (Figure 1) (Table 1). Currently, there are at least four preparations reported in clinical studies: Kenalog-40 (Bristol-Myers Squibb, Princeton, NJ, USA); preservative-free triamcinolone acetonide from compounding pharmacies; Triesence (Alcon, Fort Worth, TX, US); and Trivaris (Allergan, Irvine, CA, USA).

The Diabetic Retinopathy Clinical Research Network (DRCR) protocol B compared two doses (1 and 4 mg) of IVTA versus photocoagulation for DME [22]. For most patients, photocoagulation produced more favorable outcomes than did IVTA at 24 months of followup. Similar results were reported at 3-year followup [23]. The most common complications of IVTA are cataract formation [24] and increased intraocular pressure (IOP) [25]. Pseudoendophthalmitis [26] and infectious endophthalmitis occur much less commonly. The rate of infectious endophthalmitis after IVTA is low in reported series. For example, in an analysis of two large RCTs (from the DRCR network and the Standard Care Versus Corticosteroid for Retinal Vein Occlusion (SCORE) trials), the rate of endophthalmitis after IVTA was 0.05% [27].

A triamcinolone-eluting intravitreal implant (I-vation, SurModics, Inc., MN, USA) for the treatment of DME was suspended in a phase 2b RCT after the publication of the DRCR network results showing a benefit of laser photocoagulation over IVTA in treatment of DME [22].

In order to reduce the risk of complications associated with IVTA, the use of peribulbar triamcinolone was investigated. In a single-center, prospective trial, peribulbar triamcinolone demonstrated lesser efficacy than did IVTA [28]. The DRCR reported that peribulbar triamcinolone did not significantly benefit patients with mild DME and visual acuity of 20/40 or better [29].

To reduce the need for repeated intravitreal injections, several extended-release corticosteroid delivery systems have been studied. A fluocinolone-acetonide- (FA-) eluting intravitreal implant (Retisert, Bausch and Lomb, NY, USA) has received FDA approval for the treatment of chronic, noninfectious posterior segment uveitis [30]. This is a nonbiodegradable device that releases 0.59 *μ*g/day of FA into the vitreous cavity. It must be implanted in an operating room or similar setting. In an RCT, the effects of the device versus photocoagulation for DME were studied. At one year, DME was resolved by clinical examination and optical coherence tomography (OCT) in 57% of patients with the FA implant versus 20% of patients with photocoagulation. There were no statistically significant differences in final visual acuity between the two groups [31]. At 3 years, patients randomized to receive the FA implant had persistent treatment of macular edema, but 95% of phakic eyes developed significant cataract, and about one-third of eyes had IOP above 30 mm Hg [32].

A smaller fluocinolone acetonide-eluting device (Iluvien, Alimera Sciences, Alpharetta, GA, USA) may be administered through a 25-gauge device in a clinic setting. The Famous (Pharmacokinetic and Efficiency Study of Fluocinolone Acetonide Inserts in Patients with DME) study compared 0.2 versus 0.5 *μ*g/day fluocinolone injection devices in patients with persistent DME despite at least one previous focal/grid laser therapy [33]. There was a mean improvement of 5 letters in visual acuity in both groups at 3-month followup. The number of patients in the trial was too small to determine whether there were clinically meaningful differences in results obtained from the two doses.

The Fluocinolone Acetonide for Macular Edema (FAME) study comprised 2 phase 3 RCTs assessing the efficacy and safety of 0.2 *μ*g/day (low dose) and 0.5 *μ*g/day (high dose) inserts in patients with DME with persistent edema despite at least one macular laser treatment [34]. The primary study endpoint was defined as improvement in visual acuity by 15 or more letters at 2 years. At 24 months, the primary endpoint was achieved in 28.7% and 28.6% of low- and high- dose insert groups compared with 16.2% in the sham group. Elevated intraocular pressure requiring incisional surgery occurred in 3.7%, 7.6%, and 0.5% of the low-dose, high-dose, and sham groups, respectively.

The dexamethasone drug delivery system (DDS) [Ozurdex, Allergan, Irvine, California] is a biodegradable, sustained-release device approved by the US FDA for the treatment of macular edema associated with retinal vein occlusion and noninfectious posterior segment uveitis. A phase 2 RCT in patients with persistent macular edema secondary to various etiologies, including DME, showed that the dexamethasone DDS produced improvements in visual acuity, macular thickness, and fluorescein leakage that were sustained for up to 6 months [35]. In an RCT, the safety and efficacy of the dexamethasone DDS in the treatment of DME was studied [36]. Patients with persistent macular edema (at least 90-day duration) were randomized to treatment with 700 *μ*g or 350 *μ*g of dexamethasone DDS or observation. At 3 months, visual acuity improved by 10 letters or more in 30% of eyes in the 700 *μ*g group, 20% of eyes in the 350 *μ*g group, and 12% of eyes in the observation group. A more recent study reported that the dexamethasone DDS improved visual acuity and macular edema in previously vitrectomized eyes with diffuse DME [37].

### 2.2. Vascular Endothelial Growth Factor Antagonists

VEGF appears to play an important role in the pathogenesis of diabetic retinopathy [38]. In animal models, injection of VEGF causes breakdown of the BRB [39], and elevated levels of VEGF cause macular edema [40]. An oral nonselective blocker of VEGF receptor was found to reduce DME, suggesting that VEGF antagonists may provide benefit in treatment of DME [41]. Four intravitreal anti-VEGF agents are currently available commercially, although none is FDA-approved for the treatment of DME (Table 2).

#### 2.2.1. Pegaptanib

Pegaptanib (Macugen, Eyetech Pharmaceuticals, Palm Beach Gardens, FL, USA) is a pegylated aptamer that targets the VEGF_165_ isoform. Pegaptanib is approved by the FDA for the treatment of neovascular age-related macular degeneration (AMD) and was the first anti-VEGF medication reported to have efficacy in the treatment of DME. The Macugen Diabetic Retinopathy Study Group conducted a phase 2 RCT of pegaptanib for fovea-involving DME [42]. After 36 weeks of followup, the pegaptanib-treated eyes had better visual acuity, more reduction in central retinal thickness, and less need for laser photocoagulation compared to the sham group. More recently, a phase 2/3 RCT reported that pegaptanib therapy was associated with improved visual outcomes in patients with DME for up to 2 years [43].

#### 2.2.2. Bevacizumab

**Figure 2:**
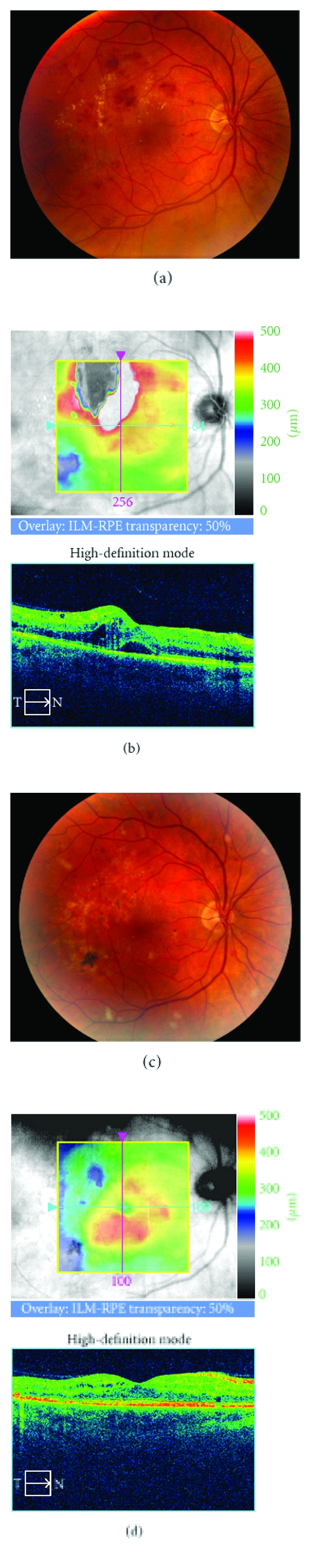
(a) Fundus photograph, right eye, of a patient with persistent diabetic macular edema following focal/grid photocoagulation. (b) Spectral domain optical coherence tomograph, right eye, demonstrates cystoid macular edema and subretinal fluid. (c) Following additional focal/grid photocoagulation and treatment with intravitreal bevacizumab, 1.25 mg in 0.1 mL, fundus photography demonstrates marked improvement in diabetic macular edema. (d) Follow-up spectral domain optical coherence tomography demonstrates marked improvement in intraretinal and subretinal fluid.

Bevacizumab (Avastin, Genentech, Inc., South San Francisco, CA, US) is a full-length recombinant humanized antibody against all isoforms of VEGF-A. Bevacizumab is approved by the FDA for the systemic treatment of metastatic colorectal cancer, metastatic breast cancer, and nonsmall cell lung cancer [44]. The agent is used commonly as an off-label intravitreal injection (Figure 2). The DRCR network conducted a randomized study of 121 eyes with DME over a 12-week period [45]. There were five treatment arms: focal photocoagulation, 2 consecutive 1.25 mg bevacizumab injections, 2 consecutive 2.5 mg bevacizumab injections, 1.25 mg bevacizumab followed by sham injection, and combination of photocoagulation with 2 consecutive 1.25 mg bevacizumab injections. The groups that received two bevacizumab injections without laser had a significant improvement in visual acuity over the laser-only group. There were no detectable differences between the 1.25 mg and 2.5 mg doses. The single injection group had no advantage over the laser-only group. The combination of laser and bevacizumab had comparable results to the laser-only group with a trend toward worse short-term vision than eyes that received two bevacizumab injections. The DRCR is currently planning an RCT to compare bevacizumab to ranibizumab in the treatment of DME.

In the BOLT (Bevacizumab Or Laser Therapy in the Management of DME) study, repeated intravitreal bevacizumab injections were compared with modified ETDRS photocoagulation in patients with persistent DME. A total of 80 patients with center-involving DME and at least one prior photocoagulation without evidence of advanced macular ischemia were included. Patients were randomized to 2 arms: intravitreal bevacizumab (injections at baseline, 6- and 12-week followup with subsequent injections every 6 weeks based on OCT-guided retreatment protocol) or photocoagulation (at baseline with subsequent retreatment every 4 months if clinically indicated by ETDRS guidelines). At 12 months, bevacizumab had a greater treatment effect than did photocoagulation. The bevacizumab arm gained a median of 8 ETDRS letters, whereas the photocoagulation group lost a median of 0.5 ETDRS letters. Approximately 31% of patients in the bevacizumab arm versus 7.9% of patients in the laser arm gained ≥10 ETDRS letters (*P* = 0.01). The decrease in central macular thickness was significantly more in the bevacizumab group compared to the photocoagulation group [46]. There was no progression of macular ischemia in either treatment group [47].

#### 2.2.3. Ranibizumab

Ranibizumab (Lucentis, Genentech, Inc. South San Francisco, CA, USA) is a recombinant humanized monoclonal antibody fragment that binds all isoforms of VEGF-A with high affinity. Ranibizumab is FDA-approved for the treatment of neovascular AMD and retinal vascular occlusion [48–51]. The Ranibizumab for Edema of the Macula in Diabetes (READ-2) study randomized 126 eyes with DME to 3 groups: ranibizumab only (injection at baseline, months 1, 3, and 5); photocoagulation (at baseline and at 3 months if needed); combined ranibizumab and photocoagulation (photocoagulation and ranibizumab at baseline, and ranibizumab at 3 months if needed) [52]. Patients randomized to ranibizumab only showed a significantly better visual outcome at 6 months compared with the other 2 groups. For patients with data available at 6 months, improvement of 3 lines or more in vision occurred in 22% of patients in the ranibizumab-only arm, none in the photocoagulation-only arm, and 8% in combined arm. At 24 months, the study reported that intravitreal ranibizumab provided persistent treatment benefits [53].

DRCR protocol I evaluated ranibizumab and IVTA in combination with photocoagulation by randomizing patients into four arms: ranibizumab with prompt (within one week) photocoagulation, IVTA with prompt photocoagulation, sham injection with prompt photocoagulation, and ranibizumab with photocoagulation deferred for at least 24 weeks [54]. The treatment protocol included a baseline treatment followed by intravitreal study medication or sham injection retreatments every 4 weeks through the 12-week visit. After the 16-week visit, retreatment was at the investigator's discretion according to web-based predetermined criteria. Ranibizumab with prompt or deferred photocoagulation resulted in more favorable visual acuity and central macular thickness outcomes compared with photocoagulation alone at 1 and 2 years of followup. In ranibizumab-treated eyes, the results were similar whether photocoagulation was given with the first injection or deferred for at least 24 weeks. IVTA combined with photocoagulation did not result in better visual outcomes compared with photocoagulation alone. In pseudophakic eyes, the IVTA with prompt photocoagulation group had similar visual outcomes to the 2 ranibizumab groups, suggesting that cataract formation may have affected the visual acuity outcomes in phakic eyes treated with IVTA. Two-year visual outcomes were similar to 1-year results and reinforced the conclusion that ranibizumab with prompt or deferred photocoagulation should be considered for patients with vision impairment of worse than 20/32 secondary to DME [55]. This study utilized a web-based algorithm to determine treatment decisions; in clinical practice, this may not be feasible, but the general approach may be emulated [56].

The RESTORE phase 3 study reported that ranibizumab monotherapy or combined with laser photocoagulation provided superior visual acuity gain over standard photocoagulation in the treatment of DME [57]. The one-year results showed that 37% of patients treated with ranibizumab 0.5 mg alone, and 43% of those treated with ranibizumab plus laser therapy, gained vision improvement of 10 letters or more compared to 16% of patients treated with laser alone. At one year, no difference was detected between the ranibizumab and ranibizumab plus laser arms.

A recent study showed that the addition of one IVTA injection or two ranibizumab injections to eyes receiving focal laser treatment for DME and panretinal photocoagulation is associated with significantly better visual acuity and decreased macular edema by 14 weeks [58]. However, these improvements were not maintained when study subjects were followed for 56 weeks for safety outcomes.

Two additional phase 3 RCTs (RISE and RIDE) were conducted to evaluate the efficacy, durability, and long-term safety of monthly ranibizumab injections in patients with center-involving DME. The primary efficacy outcome was the proportion of subjects who gained more than 15 letters in visual acuity compared with baseline at 24 months. Patients were randomized 1 : 1 : 1 to receive monthly injections of 0.3 mg ranibizumab, 0.5 mg ranibizumab, or sham. These studies were not designed to compare the two doses of ranibizumab, but each dose against the sham injection. At 24 months, RISE met its primary endpoint with statistically significant improvements in vision in ranibizumab-treated patients compared to sham injections [59].

The safety and efficacy of 2 concentrations of intravitreal ranibizumab in the treatment of DME were compared in the RESOLVE phase 2 trial [60]. Subjects were randomized to receive 3 monthly injections with either 0.3 or 0.5 mg ranibizumab or placebo. Treatment was then administered on an as-needed basis, depending on the response to initial treatment. If edema persisted, then the dose of ranibizumab was doubled after 1 month. Photocoagulation after 3 injections was given if needed. When the pooled data from the double-dose ranibizumab group (*n* = 77) were compared with the sham group (*n* = 32), there were statistically significant improvements in vision and central macular thickness.

In contrast to intravitreal corticosteroids, cataract progression associated with intravitreal VEGF antagonists has not been identified. Some patients do sustain IOP elevation following repeated injections of VEGF antagonists [61], but this effect does not appear to be as strong as that associated with intravitreal corticosteroids. In most published series, the rate of endophthalmitis following treatment with intravitreal anti-VEGF injections is about 0.03% per injection [62–64]. The incidence and severity of systemic and ocular adverse events that are associated with repeated intravitreal injections of two doses of ranibizumab (0.5 mg versus 2.0 mg) in subjects with DME are being investigated in READ-3 study.

#### 2.2.4. Aflibercept

Aflibercept, or VEGF trap-eye, (Eylea, Regeneron, Tarrytown, NY, USA), is a recombinant fusion protein with activity against all VEGF-A isoforms and PlGF that is FDA-approved for the treatment of neovascular AMD and has been shown to have short-term efficacy in the treatment of DME [65]. The DA-VINCI study assessed the efficacy and safety of intravitreal aflibercept versus laser photocoagulation in the treatment of DME. Patients were randomized to one of the following treatment arms: 0.5 mg aflibercept every 4 weeks, 2 mg aflibercept every 4 weeks, 2 mg aflibercept every 8 weeks, 2 mg aflibercept as needed, or photocoagulation. At 24 weeks, the mean change in BCVA for aflibercept arms ranged from +8.5 to +11.4 letters compared to the mean change of +2.5 letters in the laser-treated eyes (*P* < 0.01). There was no statistical significant difference between the aflibercept arms. Anatomic effects (mean change in central retinal thickness) ranged from −127 *μ*m to −195 *μ*m in aflibercept arms compared to −68 *μ*m in laser-treated eyes at 24 weeks (*P* < 0.01). At 52 weeks, the mean change in BCVA for aflibercept arms ranged from +9.7 to +13.1 letters compared to the mean change of −1.3 letters in the laser-treated eyes (*P* < 0.01) [66]. In this study population, intravitreal aflibercept produced significant improvements in visual acuity and retinal thickness as compared to laser photocoagulation at both 24 and 52 weeks. At this time, aflibercept is not approved by the US FDA for the treatment of DME.

### 2.3. Vitreolysis

The vitreous has been implicated as a cause of DME by several mechanical and physiological mechanisms, including macular traction and concentration of vasopermeable factors in the macular region [67]. A recent prospective trial by DRCR network evaluated visual and anatomical outcomes of pars plana vitrectomy (PPV) without concomitant cataract surgery for DME in eyes with moderate vision loss and vitreomacular traction. Retinal thickening was improved in most eyes, but visual acuity results were less consistent with improvement of ≥10 letters in 38%, and worsening by ≥10 letters in 22% at 6 months [68]. In a subsequent analysis, the DRCR reported that better visual outcomes were associated with worse baseline visual acuity and in eyes in which an epiretinal membrane was removed [69].

Enzymatic vitreolysis with or without PPV has been studied in the treatment of DME. Intravitreal hyaluronidase (Vitrase, ISTA Pharmaceuticals, Irvine, CA, USA) has shown evidence of safety and efficacy in reducing vitreous hemorrhage secondary to different etiologies, including proliferative diabetic retinopathy (PDR), although it has not received FDA approval for this indication [70, 71].

Induction of a posterior vitreous detachment (PVD) may be beneficial in the treatment of DME [72]. Enzymes that may have efficacy in creating a PVD include hyaluronidase, plasmin, chondroitinase, and dispase [73]. Autologous plasmin has been used by itself or as adjunct to PPV in the treatment of DME [74, 75]. Microplasmin is a recombinant human protein derived from the yeast *Pichiapastoris*. It is a truncated form of the human protein plasmin with intact protease activity. The Microplasmin Intravitreous Injection (MIVI) trial was a phase 2 RCT that evaluated the safety and efficacy of intravitreal microplasmin in facilitating the creation of a total PVD in patients scheduled for PPV [76]. The study showed that microplasmin injection at a dose of 125 *μ*g led to a greater likelihood of induction of PVD than placebo. Patients receiving microplasmin were significantly more likely to have resolution of vitreomacular traction and not to require PPV.

### 2.4. Other Ocular Agents

Other ocular agents have been studied as treatments for DME (Table 3). Animal models have demonstrated an important role for inflammation in diabetic retinopathy [77]. In early stages of diabetic retinopathy, there is upregulation of cyclo-oxygenase-2 (COX-2) that leads to elevated prostaglandin production and increased expression of VEGF with increased risk of vascular leakage and retinal neovascularization [78]. High doses of aspirin and intermediate doses of COX-2 inhibitors (celecoxib) have shown to be beneficial in early stages of experimental diabetic retinopathy [79]. Periocular celecoxib-containing microparticles have shown to inhibit elevation of VEGF for as long as 60 days in animal models [80]. A recent multicenter clinical trial failed to show any visual function benefits with celecoxib treatment in DME, although, there was a suggestive effect of celecoxib in reducing fluorescein leakage [81].

Nepafenac (Nevanac, Alcon, Ft. Worth, TX, USA), an FDA-approved topical nonsteroidal anti-inflammatory drug (NSAID), is a prodrug that is converted to amfenac in the anterior chamber [82]. In a pilot study, nepafenac has shown some efficacy in the treatment of DME [83]. The DRCR is currently beginning a phase 2 RCT studying the use of topical nepafenac to treat nonclinically significant DME.

Etanercept (Enbrel, Amgen, Inc. Thousand Oaks, CA, USA and Wyeth, Madison, NJ, USA), a recombinant fusion protein with activity against TNF-*α*, is FDA-approved for the treatment of psoriatic disease [84]. Intravitreal etanercept has shown some evidence of efficacy against refractory DME [85].

Infliximab (Remicade, Centocor, Horsham, PA, USA) is another TNF-*α* antagonist that is FDA-approved for the treatment of Crohn's disease [86]. A pilot study showed benefits from systemic infliximab in treatment of DME [87]. A pilot study of intravitreal infliximab is continuing.

Stimulation of nicotinic acetylcholine (nACh) receptors on vascular endothelial cells promotes angiogenesis and vascular permeability in animal models [88, 89]. A recent multicenter phase 1/2 clinical trial evaluated the safety and bioactivity of topical mecamylamine, an antagonist of nACh receptors, in patients with DME [90]. Mecamylamine drops were well tolerated. The study suggested that administration of topical mecamylamine may have heterogeneous effects in patients with DME. The heterogeneous response may be secondary to variable expression of nACh receptor subtypes on endothelial cells.

A pilot study has reported a short-term positive response to intravitreal erythropoietin in a group of patients with chronic DME unresponsive to other therapies [91].

## 3. Systemic Agents

Various systemic agents have been studied in the treatment of DME (Table 4). Activation of protein kinase C (PKC) may play an important role in the development and progression of diabetic retinopathy [92–98]. Ruboxistaurin (Arxxant, Eli Lilly and Company, Indianapolis, IN, USA) is a selective antagonist of PKC *β*I and PKC *β*II [99]. The PKC-Diabetic Retinopathy Study (PKC-DRS) reported that ruboxistaurin was associated with a reduced incidence of moderate visual loss (doubling of the visual angle) [100]. The PKC-DRS 2 reported that ruboxistaurin was associated with a reduced incidence of sustained moderate visual loss (for 6 months) [101]. The PKC-DME Study (PKC-DMES) reported some evidence that ruboxistaurin was associated with reduced progression of DME, although this was a secondary endpoint [102]. Ruboxistaurin has not received approval from the USA FDA.

Fenofibrate is a fibric acid derivative with pleiotropic effects that is used as a lipid-modifying agent [103]. The fenofibrate intervention and event lowering in diabetes (FIELD) study, a large RCT, showed that treatment with fenofibrate reduces the need for laser treatment in patients with PDR and DME [104].

Rosiglitazone (Avandia, GlaxoSmith Klein, Research Triangle Park, NC, US) is a peroxisome proliferator-activated *γ* ligand that is used in the treatment of type 2 diabetes [105]. Treatment with rosiglitazone has been shown to reduce the rate of progression to PDR [106], however, in some patients, it may be associated with increased risk of DME [107].

## 4. Summary Statement

For decades, standard treatments for DME have included tighter control of systemic metabolic factors, as well as photocoagulation. However, some patients continue to lose vision despite these therapies, which has led to the investigation of various pharmacotherapies for DME. At this time, both intravitreal corticosteroids and intravitreal anti-VEGF agents are widely used in clinical settings. The role of combination therapies (both various medications with each other as well as medications with photocoagulation) is yet to be determined. As we continue to collect data from current and future RCTs, management strategies for DME will continue to evolve.

## Figures and Tables

**Table 1:** Selected clinical trials of corticosteroids in treatment of diabetic macular edema.

Agent (no. patients)	Main outcomes	Reference
Intravitreal triamcinolone (693)	Less favorable outcomes versus photocoagulation at 24 and 36 months	[22, 23]
Peribulbar triamcinolone (109)	Less favorable outcomes versus intravitreal triamcinolone at 34 weeks	[28]
Fluocinolone acetonide implant (Retisert) (197)	Effective treatment of DME at 36 months, but high risks of cataract and glaucoma	[32]
Fluocinolone acetonide implant (Iluvien) (956)	Generally favorable results at 24 months	[34]
Dexamethasone drug delivery system (Ozurdex) (171)	Generally favorable results at 90 days	[36]

**Table 2:** Selected clinical trials of VEGF antagonists in treatment of diabetic macular edema.

Agent (no. patients)	Main outcomes	Reference
Pegaptanib (260)	More favorable outcomes versus sham at 2 years	[43]
Bevacizumab		
DRCR phase II (121)	More favorable outcomes versus photocoagulation at 3 weeks	[45]
BOLT study (80)	More favorable outcomes versus photocoagulation at 1 year	[46]
Ranibizumab		
READ-2 study (126)	More favorable outcomes versus photocoagulation at 2 years	[53]
DRCR protocol I (691)	Ranibizumab with photocoagulation more favorable than photocoagulation alone at 2 years	[54]
RESTORE study (345)	Ranibizumab with or without photocoagulation more favorable than photocoagulation alone at 1 year	[57]
RISE/RIDE studies (377)	More favorable outcomes versus sham at 2 years	[59]
RESOLVE study (151)	More favorable outcomes versus sham at 1 year	[60]
Aflibercept		
DA VINCI study (219)	More favorable outcomes versus photocoagulation at 1 year	[66]

**Table 3:** Selected Other Ocular Agents in Treatment of Diabetic Macular Edema.

Agent (# patients)	Main Outcomes	Reference
Celecoxib (86)	Unfavorable outcomes versus photocoagulation at 2 years	[81]
Nepafenac (1)	Some evidence of efficacy in case report	[83]
Etanercept (7)	Some evidence of efficacy in pilot study	[85]
Infliximab (4)	Some evidence of efficacy in pilot study	[87]
Mecamylamine (23)	Some evidence of efficacy in pilot study	[90]

**Table 4:** Selected Systemic Agents in Treatment of Diabetic Macular Edema.

Agent (# patients)	Main Outcomes	Reference
Ruboxistaurin (686)	Did not meet primary outcome measure at 30 months	[102]
Fenofibrate (9795)	Favorable outcomes versus placebo at average of 5 years	[104]
Rosiglitazone (30)	Some evidence of efficacy at 3 months, but also may worsen DME in some patients	[107]
